# HTLV-I p30 inhibits multiple S phase entry checkpoints, decreases cyclin E-CDK2 interactions and delays cell cycle progression

**DOI:** 10.1186/1476-4598-9-302

**Published:** 2010-11-23

**Authors:** Hicham H Baydoun, Joanna Pancewicz, XueTao Bai, Christophe Nicot

**Affiliations:** 1 University of Kansas Medical Center. Department of Pathology and Laboratory Medicine, Kansas City, 66160 KS USA

## Abstract

**Background:**

Human T-cell leukemia virus type I (HTLV-I) has efficiently adapted to its host and establishes a persistent infection characterized by low levels of viral gene expression and slow proliferation of HTLV-I infected cells over decades. We have previously found that HTLV-I p30 is a negative regulator of virus expression.

**Results:**

In this study we show that p30 targets multiple cell cycle checkpoints resulting in a delayed entry into S phase. We found that p30 binds to cyclin E and CDK2 and prevents the formation of active cyclin E-CDK2 complexes. In turn, this decreases the phosphorylation levels of Rb and prevents the release of E2F and its transcriptional activation of genes required for G1/S transition. Our studies also show that HTLV-II p28 does not bind cyclin E and does not affect cell cycle progression.

**Conclusions:**

In contrast to HTLV-I, the HTLV-II-related retrovirus is not oncogenic in humans. Here we report that the HTLV-I p30 delays cell cycle progression while its homologue, HTLV-II p28, does not, providing evidence for important differences between these two related retrovirus proteins.

## Background

Cell cycle progression from G1 to S phase is regulated by the sequential activation of two kinase-complexes, CDK4/6-cyclin D and Cyclin E-CDK2 [1], to ease the inhibition of E2F-mediated transcription. In G1-phase, hypo-phosphorylated Rb binds to and sequesters the E2F-DP1 transcription factors in a repressive complex containing HDAC, thereby inhibiting the activation of key downstream transcription events [2]. Following phosphorylation of Rb by Cyclin D-CDK4/6 and subsequently by Cyclin E-CDK2, E2F is released from the repressor complex Rb-E2F allowing activation of key genes required for S-phase entry [3].

Unlike the cyclin D-dependent kinases, the activity of cyclin E-Cdk2 is intermittent and reaches a maximum at the G1- to S-phase transition [4-6]. Cyclin E expression and activity is at least in part mitogen-dependent, and its downstream targets include a subset of the G1 inhibitors that are also targeted by the D-type cyclins, Rb and p27Kip1. However, the mechanisms by which cyclin E inactivates these inhibitors differ from those used by cyclin D-dependent kinases, suggesting that their actions may be complementary [7,8]. Cyclin E-Cdk2 phosphorylates Rb on different sites from the cyclin D-dependent kinases, and may differentially affect interactions of Rb with E2Fs, histone deacetylases, and other chromatin-remodeling factors [9]. The functions of cyclin E-Cdk2 are not strictly limited to G1. Cyclin E-Cdk2 phosphorylates a second set of substrates that are involved in cell duplication; these events affect histone gene expression, centrosome duplication, replication origin licensing, and, possibly, origin firing [10]. Cyclin E is one of the E2F-responsive genes. Once the E2F transcriptional program is initiated and sufficient levels of cyclin E-dependent Cdk2 activity is attained, cells no longer rely on the cyclin D-dependent kinases nor on persistent mitogenic signals and are committed to complete the cell cycle [11].

Human T-cell leukemia virus type I (HTLV-I) was originally isolated from a patient with cutaneous T-cell lymphoma [12]. HTLV-I is the causative agent of adult T-cell leukemia (ATL) [13] and tropical spastic paraparesis/HTLV-associated myelopathy (TSP/HAM) [14,15]. HTLV-I associated malignancies are characterized by an excessive proliferation of HTLV-I infected T cells [16]. Numerous studies have reported the ability of Tax to target cell cycle checkpoints [17-23]. However, recent studies also suggest that infection with HTLV-I or Tax expression itself may not be sufficient for a sustained active cellular proliferation and that accumulation of genetic defects may be required to bypass cell cycle checkpoints [24-26]. This would in fact explain the ability of HTLV-I transformed cells to proliferate in vivo in the absence of most viral gene expression. Additional studies also showed that several virus-encoded genes, p13, p30, p12 and HBZ, adversely affect cell cycle progression [27-34].

We previously demonstrated that p30 is a post-transcriptional repressor of HTLV-I replication [35]. Additional observations suggested that p30 is a multifunctional protein that selectively regulates cellular and viral gene expression and delays infected cells in their progression to the G2 phase of the cell cycle [28,29,36-40]. In the present study, we show that HTLV-1 p30 delays the cell cycle before the entry into S phase. We also show that the effect of p30 is due to its interaction with the cyclin E key-trigger of the G1/S transition, which in turn reduces the function of the Cyclin E-CDK2 complex and all the downstream events.

## Methods

### Plasmids and lentiviral particles

Lentiviral particles expressing p30-myc or GFP were prepared by transfection of 293FT cells with HR-CMV-p30myc or GFP with pDLN and VSV-G, respectively, as previously reported [35]. The genes encoding for HTLV-I p30 and its homologue, HTLV-II p28, proteins were amplified by PCR and cloned in frame with an HA tag of pMH vectors into the HindIII and EcoRI sites. The same sites were used to clone both genes in frame with GFP in pEGFPC1 (clonetech). Cyclin E-myc and CDK2-HA expression vectors were a gift from James M. Roberts [41].

### Cell Culture and transfection

Hela cells and 293FT were obtained from the ATCC (American Type Culture Collection). They were maintained in Dubelcco modified Eagle medium, DMEM, complemented with 10% of fetal bovine serum (Gibco) and 1% penicillin-streptomycin. Jurkat T cells were cultured in RPMI complemented with 10% of fetal bovine serum (Gibco) and 1% penicillin-streptomycin. Cells were maintained at 37°C in a humid incubator with 5% CO2 concentration. Polyfect transfection reagent (Qiagen) and Calcium phosphate complexes (Invitrogen) were used to transfect HeLa and 293FT cells, respectively. The luciferase reporter (pGL3) whose expression is driven by six E2F responsive elements (6xE2F-Luc) [42] was used for the transcriptional activity of E2F proteins. 293T cells were transfected with 6xE2F-Luc along with increased amounts of HTLV-1 p30 or HTLV-2 p28 expression vectors. After 24 h of transfection, the cells were starved overnight with media containing 0.5% FBS and then were serum activated (15% FBS) for 5 h followed by luciferase activity assays of the cell extracts as previously reported [42].

### Cell cycle and Flow Cytometry analyses

For cell cycle synchronization and release, 10 h after transfection (GFP vectors), cells were treated overnight with Hydroxyurea (2 mM) to arrest cells in the G1 phase of the cell cycle. They were next washed and released from block for the time indicated in the figure legends. For the accurate counting of cells going through the cell cycle, in some experiments cells were treated with Nocodazole (40 ng/ml) after release from Hydroxyurea to block them in G2/M. For the cell cycle distribution analysis, cells were resuspended in media containing the Dye Cycle Violet (Excitation at 405 nm and Emission at 450 nm) (Invitrogen) and incubated for 30 min at 37°C before being analyzed by an LSRII flow cytometer using FACS DIVA 6.1 software.

### Immunocytofluorescence staining and microscopy

GFP or p30-myc expressing viruses have been titered by immuno-fluorescence to reach 100% of cells transduced. For immunostaining, Hela cells seeded on coverslips were transduced for 48 Hours with the viral particles expressing p30-myc. They were then fixed in 3.7% Paraformaldehyde (PFA) for 15 min at RT, washed with PBS for 5 min followed by permeabilization with 0.5% Triton X-100 on ice for 5 min. They were next blocked for 1 h in PBS with 0.5% gelatin and 0.25% bovine serum albumin at room temperature. Cells were incubated overnight with anti-myc primary antibody (9E10, Roche), washed 3 times with PBS plus gelatin 0.2% and then incubated with anti Mouse Alexa-596 conjugated secondary antibody (Invitrogen). They were next washed and mounted by using DABCO mounting medium (2.5% DABCO from Sigma, 200 mM Tris-HCl pH8.6 and 90% glycerol). Fluorescent images were captured by using a Nikon T*i*-S epifluorescence microscope equipped with a device camera and the NIS elements software (Nikon). The images were collected by using the objectives 20× and 100×.

### RNA extraction and Real time RT-PCR

The expression of E2F, cyclinE and p21 was quantified by real time PCR using the Sybr Green method (Applied Biosystems). Briefly, the total mRNA of cells transfected or not transfected with the p30 expression vector was extracted by Trizol, DNase-I treated and reverse-transcribed using the High capacity RNA to cDNA kit (Applied Bioscience) as recommended by the manufacturer. Real time PCR (40 cycles) was performed in a total of 25 ul containing RT master mix, water, primers at 0.5 uM each and cDNA as recommended by the manufacturer using Applied Bioscience Step One plus real time PCR Software. The GAPDH gene was used as an internal amplification control. GAPDH primers: Forward 5'-gaaggtgaaggtcggagtc-3' and Reverse 5'-gaagatggtgatgggatttc-3'. Real time primers: cyclinE forward: tcagggtatcagtggtgcga, cyclinE reverse: caaatccaagctgtctctgtg, p21 forward: tgcgttcacaggtgtttctg, p21 reverse: gccattagcgcatcacagt, E2F forward: accctgacctgctgctctt and E2F reverse: tctcggccaggtactgatg. The results are determined by the Relative Standard Curve and comparative C_T _Experiments. The fold = 2^-DDCT.^

### Immunoprecipitation, western blots and antibodies

Cell extracts were prepared using NP-40 lysis buffer (1% NP-40, 50 mM Tris-HcL (pH7.5)), 150 mM NaCl, and a set of proteases (inhibitors). The immunoprecipitation was performed overnight and the proteins were separated by SDS page, transferred to PVDF membrane and western blotted using the standard protocol of WB: Blocking with TBS/Milk 5%, primary antibody overnight incubation and secondary antibody 2 h incubation (in TBS Milk 2%), washed 3 times in TBST 0.05% and signals were revealed with super signal chemiluminescent substract (Thermo Scientific). Primary antibodies used in this study: Rabbit polyclonal anti phospho(Thr821/826)-Rb (Santa Cruz), Goat polyclonal anti Actin sc-1615 (Santa Cruz), Rabbit polyclonal anti cyclinE (Santa Cruz C-19 clone), Mouse monoclonal anti-myc tag (9E10) and Mouse monoclonal anti-HA (3F10), Mouse monoclonal anti Flag (Roche), goat polyclonal anti Rb sc-50-G (Santa Cruz), Rabbit polyclonal anti E2F-1 sc-193 (Santa Cruz), Mouse monoclonal anti PCNA (pc10, ZYMED) and Rabbit polyclonal anti p21waf sc-397 (Santa Cruz).

## Results

**Figure 1:**
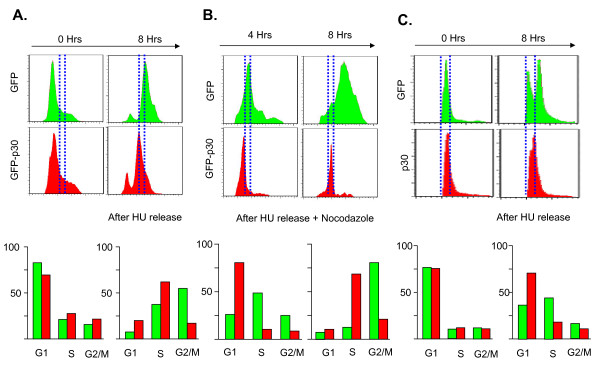
**HTLV-1 p30 delays entry of cells into S phase of the cell cycle**. Hela cells were transfected with GFP (green) control or GFP-p30 (red) expression vectors synchronized with Hydroxyurea (2 mM) and then released for 0 h or 8 h **A**). **B**) The same experiment was performed as in A) but after release from "HU" block, cells were immediately treated for 4 h or 8 h with Nocodazole (40 ng/ml) to prevent the reentry of cells from G2/M to G1. The cell cycle was assessed by flow cytometry of GFP positive gated cells. Histograms indicating the percentage of cells in each phase of the cell cycle shown in **A**) and **B**), respectively. **C**) Jurkat cells infected with lentiviral particles expressing p30 or GFP were synchronized overnight with HU (2 mM) and then released for 0 h and 8 h. Cells were stained with propidium iodide (PI) and analyzed by Flow cytometry for the cell cycle distribution using the FACS DIVA 6.1 and FlowJo7.5 Softwares.

Using genome-wide microarray analyses, we and others have previously shown that HTLV-I regulatory protein p30 modulates a number of genes involved in the cell cycle. In addition, p30 has been shown to promote the accumulation of cells in the G2-M phase of the cell cycle to facilitate early viral spread and increase T-cell survival following infection [29]. Since our previous data suggested that p30 alters expression of genes involved in G1/S progression [28], we investigated the possible role of p30 in early phases of the cell cycle. To facilitate analyses of p30 expressing cells by FACS, p30 was cloned into a vector containing an internal ribosome entry site (IRES) fused to the green fluorescent protein (GFP). Cells transfected with GFP-p30 or GFP vector were treated overnight with Hydroxyurea (HU) in order to synchronize cells in the G1 phase of the cell cycle (Figure 1A). Approximately, 75% of p30-expressing or GFP control cells were successfully blocked in G1, and the percentage of p30-expressing or GFP control cells in S and G2/M were similar (Figure 1A). Cells were washed, released from arrest for eight hours before the cells were collected, stained with DyeCycle violet and the GFP expressing cells gated by FACS for cell cycle analyses. Interestingly, while the majority of GFP expressing cells progressed to G2/M phase (green), p30-expressing cells were significantly delayed and were still mostly in S phase (red). Since HU did not block 100% of the cells and to prevent the reentry of cells from G2/M to G1, we repeated this experiment in the presence of nocodazole, an inhibitor of the mitotic spindle formation that promotes arrest of Jurkat cells in G2/M [43]. In this experiment the initial time point prior to release is the same as in Figure 1A. The results presented above suggested that p30-expressing cells were delayed in G1/S progression and/or during DNA replication in S phase. To differentiate between these two possibilities, we analyzed cells four hours after release. Data indicated that p30-expressing cells were still in G1/S (80%) while GFP control cells already entered S phase, about 50% for only 10% of p30-expressing cells (Figure 1B). These results suggest that p30-expressing cells are mainly delayed at the G1/S border and their entry into S phase. Our results also suggest that p30 delays, but does not block cell cycle progression, since eight hours after release most p30-expressing cells are in S phase (68%)(Figure 1B). Again, while most GFP expressing cells are in G2/M (80%) eight hours after release, only about 20% of p30-expressing cells are in G2/M (Figure 1B). Collectively, our results demonstrate that p30 prevents normal cell cycle progression, retains cells in G1 and favors a cellular resting state. To confirm our results in human T cells, the natural target of HTLV-I, we used lentiviral vectors to tansduce p30 or GFP into Jurkat. Twenty four hours after infection, cells were synchronized with HU and released for cell cycle progression at time t = 0 and t = 8 hours. The results presented in Figure 1C further demonstrate that p30 transduced Jurkat cells were significantly delayed in S-phase entry when compared to GFP transduced Jurkat cells.

**Figure 2:**
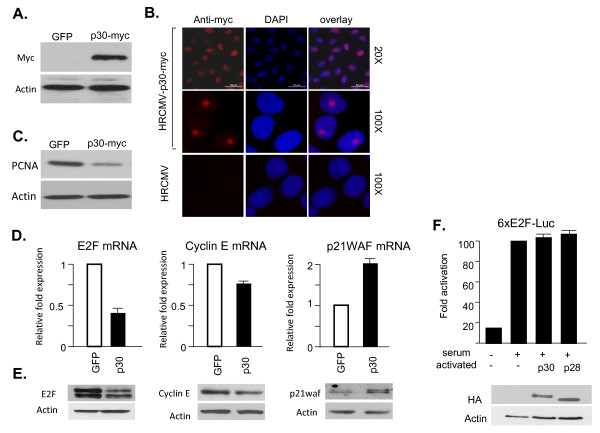
**p30 decreases the expression of proteins involved in S phase entry and progression**. Hela cells were transduced for 48 h with GFP (control) or p30-myc expressing lentivirus. **A**) p30-myc expression was detected by western blot (WB) using anti-myc antibody or **B**) by indirect immunofluorescence (IF) staining. Nearly 100% of cells were transduced as shown with different magnifications. GFP-viral particles (HRCMV-GFP) were used as a control in the IF staining. **C**) WB analysis of PCNA protein was performed in the same experimental conditions. **D**) Quantification of E2F, cyclin E and p21waf mRNAs by real time PCR. E2F, Cyclin E and p21waf values were normalized to that of GAPDH which was used as an internal control. **E**) Extracts of Hela cells transduced with GFP or p30-myc virus were used for WB analyses of the endogenous E2F, cyclin E and p21 proteins. **F) **293T cells were transfected with 6xE2F-Luciferase reporter plasmid either with HTLV-1 p30 or HTLV-2 p28 expression vectors. Histograms represent the values of three different experiments with their standard deviations. WB analyses showing equal amounts of p30 and p28 are present in the cell extracts.

We next wanted to study the effect of p30 on the expression of endogenous G1/S regulators. Since transfection efficiencies are not sufficient for such studies, we cloned p30 with a carboxy-terminal myc tag into a lentiviral vector. High titer pseudotype virus particles were prepared by transfection of 293FT cells with HR-CMV-p30myc, pDLN and VSV-G. A GFP expressing virus was also prepared in the same conditions, titered by immuno-fluorescence and used as control. Expression of p30 was readily detected by western blot using an anti-myc antibody forty-eight hours after infection of HeLa cells (Figure 2A), and, in our experimental conditions, nearly 100% of cells were transduced, as shown by immuno-fluorescence detection of p30 (Figure 2B). Using the above experimental conditions, we analyzed the expression of proliferating cell nuclear antigen (PCNA), a protein involved in replication of the DNA and required for entry and progression of cells in S phase. The results showed that p30 expression significantly reduced PCNA expression at the protein level (Figure 2C).

We next tested the expression of several regulators of the G1/S transition, E2F, cyclin E and histone H2A, by quantitative real time RT-PCR. Our results showed significant down-regulation of *E2F *and *cyclin E *mRNA in the presence of p30 (Figure 2D). We did not see any significant change in H2A levels (data not shown). These data were further confirmed at the protein level by western blot analyses (Figure 2E). Since E2F is a key transcriptional regulator of genes involved in S phase entry [44], decreased expression of E2F in part explains the reduced expression of PCNA and cyclin E (Figure 2C and 2D). Our studies also revealed an increased expression of p21waf CDK-inhibitor at both RNA and protein levels (Figure 2D and 2E). Although many genes, including E2F itself, are transcriptionally regulated by E2F, our results shown in Figure 2F suggested that p30 do not directly affect E2F transcriptional activity. Overall, our results suggest that p30 alters cell cycle progression by targeting multiple checkpoints involved in the G1/S transition, thereby preventing S phase entry.

**Figure 3:**
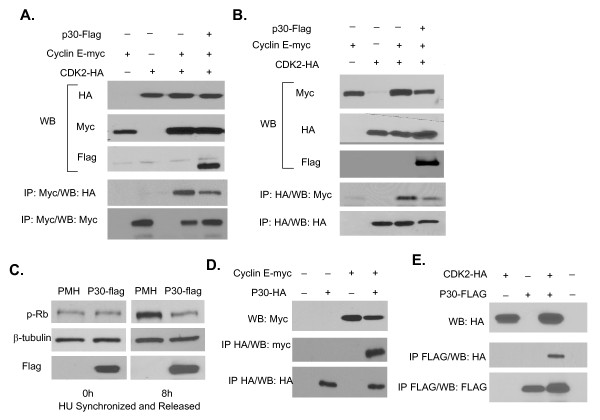
**p30 interacts with Cyclin E and CDK2 and reduces formation of the Cyclin E-CDK2 complex**. **A and B**) p30 disrupts interactions between Cyclin E and CDK2. 293T cells were tranfected with Cyclin E-myc, p30-flag and CDK2-HA. The interactions between Cyclin E and CDK2 in the presence or absence of p30 were assessed by either co-IP of Cyclin E or CDK2 and western blot for the corresponding partner. **C**) Phosphorylation of Rb protein. 293T cells were transfected with empty control vector or p30 expressing vector, synchronized with "HU" and released for 0 h and 8 h. The cells extracts were then analyzed by WB to detect phosphorylated Rb. **D**) Interactions between Cyclin E and p30 or (**E**) CDK2 and p30 were investigated after transient transfection in 293T cells and Co-IP/WB.

Whereas cyclin D/cyclin dependent kinase (CDK) 4 and 6 control early G1, progression through G1/S is controlled by the cyclin E-CDK2 complexes. In the absence of Cyclin E-CDK2 activity, E2F remains bound to Rb and cannot activate the transcription of genes involved in S phase entry. Previous studies have shown that Tax directly alters the ratio of Rb-bound and Rb-unbound E2F resulting in activation of E2F-dependent transcription and S-phase entry [45]. Activity of the cyclin E-CDK2 complexes occurs through distinct mechanisms, including activation of CDK2 by phosphorylation [46] or alterations in the stoichiometry of cyclin dependent kinase inhibitors (CDKI) p21^Waf1 ^and p27^Kip1 ^bound to cyclin E [47]. To identify which of these mechanisms may be associated with p30-mediated inhibition of G1/S progression, the active form of CDK2 bound to cyclin E was detected by immuno-precipitation of cyclin E from protein extracts of cells transfected with cyclin E-myc and CDK2-HA in the presence or the absence of p30-Flag. Our results demonstrated that the amounts of active CDK2 bound to cyclin E is severely reduced in the presence of p30 (Figure 3A and 3B). Consistent with these findings we also found that in the presence of p30, decreased amounts of active Cyclin E-CDK2 complexes correlated with reduced hyperphosphorylation of Rb p107 (Figure 3C). It is well known that in the absence of phosphorylated Rb, E2F cannot be released and cannot activate the transcription of genes required for S phase entry. We next investigated whether p30 may interact with either cyclin E or CDK2. Specific interactions between p30 and cyclin E were readily demonstrated by co-immunoprecipitation and western blot assays after transfection of 293T cells (Figure 3D). Similarly, we found that p30 forms a complex with CDK2 (Figure 3E). Our results suggest that p30 interacts with cyclin E and CDK2 reducing Cyclin E-CDK2 complex formation and phosphorylation of Rb.

**Figure 4:**
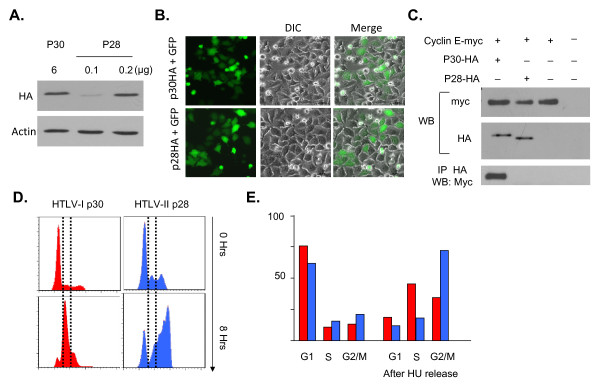
**p30 homologue HTLV-2 p28 protein does not interact with Cyclin E and does not affect cell cycle progression**. **A**) 293T cells were transfected with comparable expression amounts of plasmids expressing p30-HA and p28-HA proteins, WB with HA antibody or actin for loading control. **B) **Hela cells were transfected with p30HA or p28HA along with GFP expression vector and GFP expression was monitored to demonstrated equivalent transfection efficiencies. **C**) p28 does not interact with Cyclin E. 293T cells were transfected either with Cylin E-myc and p30-HA or cyclin E-myc and p28-HA. Cell extracts were prepared and the interaction in vivo between Cyclin E and both viral proteins were assessed by co-immunoprecipitation. **D**) p28 does not affect the cell cycle progression. Hela cells were transfected either with GFP-p30 or GFP-p28 expression vectors, synchronized with Hydroxyurea (2 mM) and then released for 0 h, 8 h. The cell cycle distribution was assessed by flow cytometry of GFP positive gated cells using the Dye cycle Violet. **E**) Histograms indicating the percentage of cells in each phase of the cell cycle distribution.

While HTLV-I is the etiological agent of ATL and TSP/HAM, infection with HTLV-II is not oncogenic in humans. We next investigated the effect of the HTLV-I p30 homologue, HTLV-II p28 [48], on cyclin E and the cell cycle. To compare p30 and p28 biological activities we cloned both viral genes into the same vector (pMH) with the same tag (HA). Following transfection of 293T cells, our results consistently showed that the HTLV-II p28 protein expressed 25 to 30 fold higher than the p30 protein (Figure 4A). This difference in levels of expression between p30 and p28 was consistently observed and was not related to differences in transfection efficiency (Figure 4B). Using normalized protein amounts, we found that, in contrast to p30, p28 was not able to bind cyclin E (Figure 4C) following transient expression in 293T cells, unless p28 was overexpressed at extremely high levels (data not shown). Finally, cell cycle analyses revealed that p28 does not stop the progress of cells in the transition from G1 to S phase as does p30 (Figure 4D and 4E), thereby providing the first evidence of a functional difference between these two proteins.

## Discussion

Because HTLV-I is highly immunogenic and presents a low variability, reducing expression of viral proteins is essential to virus maintenance *in vivo*. In fact, while HTLV-I expression can be detected in TSP/HAM patients, the virus is mainly silent in ATL patients [49] and expansion of infected cells occurs through cell division rather than de novo infection [50]. We have previously discovered that the p30 complex with the *tax/rex *mRNA prevents its nuclear export, thereby repressing virus expression [35]. This may be advantageous for the establishment of a latent and persistent infection. We also showed that p30 decreases Toll-like receptor 4 (TLR4) signaling and inhibits the production of pro-inflammatory cytokines (macrophage chemoattractant protein 1(MCP-1), tumor necrosis factor alpha (TNF-α), and interleukin 8 (IL-8)), while increasing the release of the anti-inflammatory cytokine IL-10 [38]. These findings may in part explain the inability of dendritic cells to activate adaptive immunity in ATL patients and the limited proliferation of virus-specific cytotoxic T-lymphocytes (CTL) reported in ATL patients. Previous studies have shown that p30 can delay the progression of cells during G2/M by enhancing Chk-1 phosphorylation and reducing expression of Polo-like kinase (PLK1) [29].

In the present study, we demonstrate that the HTLV-I accessory protein p30, but not its HTLV-II homologue p28, delays entry of cells into S phase and cell cycle progression. While proliferation of infected cells is required at times, uncontrolled proliferation of infected cells may lead to their activation and expression of viral genes. Therefore, it seems logical that the virus evolved proteins to balance proliferation as well as mechanisms to prevent infected cells from rapid cell division. Our results demonstrate that p30 activates multiple G1/S checkpoints to reduce proliferation. We did not detect any significant change in H2A mRNA levels of expression in p30 expressing cells. We think that this may be due to the fact that p30 targets factors involved in S phase entry and regulated by E2F. In contrast H2A activation is E2F-independent and occurs after cells have entered S phase. Cyclin-dependent kinase inhibitor (CDKI) p21waf expression was increased in p30 expressing cells. We do not know the mechanism by which p30 increases p21waf expression at the moment and additional studies are needed to investigate whether p30 affects the expression of p21waf by increasing its promoter expression or mRNA stability. Alternatively p30 may affect other cellular factors involved in p21waf expression such as p53 or Mdm2. We found a significant reduction in E2F and Cyclin E expression at both the transcriptional and protein levels. We do not think that p30 directly affects E2F promoter, since we did not see significant effects of p30 on E2F-mediated transcriptional activation of an E2F reporter construct. Instead, we found that p30 interacts with both Cyclin E and CDK2 preventing the formation of active Cyclin E-CDK2 complexes. Our data may suggest that like p21waf, p30 may form a ternary complex with these proteins further studies are needed to clarify the mechanism. Whether p30 prevents the formation of Cyclin E-CDK2 complexes by sub-cellular compartmentalization to the nucleolus or recruitment of additional cellular factors is currently under investigation. The effects of p30 on the cyclin E-CDK2 complex was associated with a significant decreased phosphorylation of Rb and delayed entry into S phase.

In contrast to a previous study [29] using a stable transfected p30 Jurkat cell line, we did not observe a significant delay of p30 expressing cells in progression through G2/M in our assays. While the systems used are quite different, it is possible that selection of p30 expressing cells in that study resulted from elimination of cells with a significant delay in G1/S entry. On the other hand it is possible that the G2/M accumulation previously reported for p30 expressing cells is in fact the result of a delay of these cells in S phase entry. Thus entry of p30 expressing cells into G2/M occurs later while control cells have already completed their cycle and are in G1. In fact, when p30 expressing and control cells were synchronized in G2/M using nocodazole and released we did not see any delayed cell cycle progression from G2/M to G1. However, it is still possible that nocodazole does not block cells early enough in G2 to expose p30 effects. Clearly additional experiments are needed to clarify these issues.

**Figure 5:**
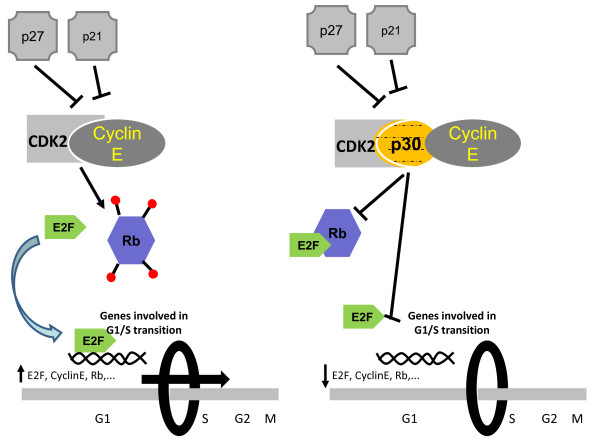
**The progression through G1/S transition in the presence or absence of HTLV-1 p30**. HTLV-1 p30 disrupts the interaction between CDK2 and Cylin E and the capacity of this complex to phosphorylate Rb. E2F becomes sequestered by Rb and is not available to activate the expression of genes involved in G1/S transition.

Together our data lead to a model (Figure 5) in which p30 prevents the release of E2F from Rb, thereby preventing E2F-mediated transcription of genes involved in the transition of G1 to S as well as its own expression. Interestingly, these effects of p30 would be predicted to antagonize those of Tax, which inactivates or overrides cell cycle checkpoints and stimulates cell proliferation. Differences between Tax and p30 are, however, not completely unexpected given the opposite functions of these two proteins during the virus life cycle. Interestingly, our data demonstrate that the HTLV-II p28 protein does not interact with cyclin E and does not significantly impair cell cycle progression. This is the first report of a physiological difference between p30 and p28. Because HTLV-I, but not HTLV-II, is associated with leukemias, any difference between these viruses may offer some clues about the determinant of viral pathogenesis of HTLV-I.

In addition to p30, other HTLV-I-encoded small proteins play a critical role in evasion of infected cells to immune defenses and survival of tumor cells. p12 has been shown to down regulate MHC class I from the cell surface and to increase the release of calcium from the endoplasmic reticulum to activate NFAT-mediated transcription [30,31,51-54]. Finally, proteolytic processing of p12 generates a shorter 8-kDa protein that migrates to the cell surface, interacts with the immunologic synapse following engagement of the T-cell receptor (TCR), and down-regulates TCR signaling [55]. p13, a mitochondrial protein, induces a delay in all phases of the cell cycle and appears to be mediated by up-regulation of a heat-sensitive cellular cytostatic factor [27]. These studies suggest that HTLV-I accessory proteins may play critical roles in slowing the replication of HTLV-I infected cells in vivo to prevent their elimination by the host's immune cells. A better understanding of how this is achieved may offer new ways to interfere with viral latency, expose infected cells to the immune system and help their eradication.

## List of abbreviations

E2F: HTLV-I; PCNA: Rb.

## Competing interests

The authors declare that they have no competing interests.

## Authors' contributions

HB performed experiments for figures 1, 2, 3, 4 and 5 and wrote the paper. JP performed experiments for figures 2 and 3. BXT performed experiments for figures 3 and 4. CN designed the study, interpreted the data and wrote the paper. All authors read and approved the final manuscript.
